# Supplementary material for: Thermogenetic neurostimulation with single-cell resolution
Source: Nat Commun. 2017 May 22;8:15362. doi: 10.1038/ncomms15362 (PMC5493594; doi:10.1038/ncomms15362)
Supplement: Supplementary Information — Supplementary Figures, Supplementary Note and Supplementary References [file ncomms15362-s1.pdf]

Supplementary figures

Supplementary figure 1

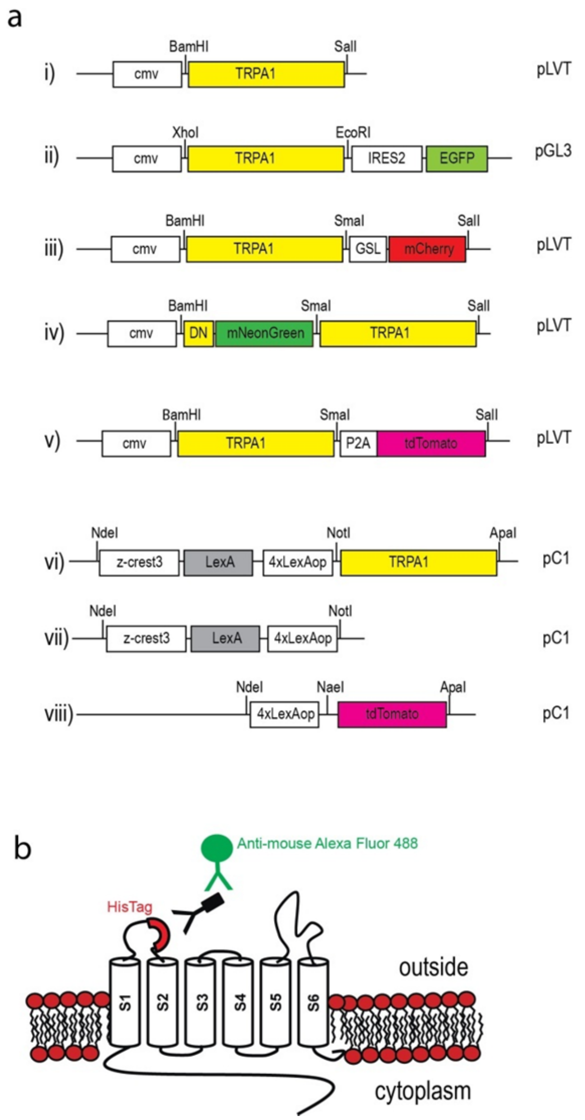

Supplementary Figure 1. TRPA1 labeling strategies. (**a**) Schemes of constructs with different methods of fluorescent labeling of TRPA1. i) wild type TRPA1, non labeled; ii) TRPA1-IRES2-EGFP; iii) C-terminal fusion protein of TRPA1 and mCherry, GSL is linker consisting of 31 amino acids poly-serine-poly-glycine tract; iv) N-fusion of mNeonGreen and TRPA1 (dN-mNeonGreen-caTRPA1), doubled N-terminal targeting sequence (a.a. 1-60) from caTRPA1 (DN) is added to the N-terminus of mNeonGreen. v) TRPA1-P2A-tdTomato, auto-cleavage site P2A enables eqimolar concentrations of TRPA1 and tdTomato. vi) tissue specific construct for TRPA expression in Zebrafish larvae somatosensory neurons. This construct was used along with vii) and viii) that upon co-transfection produce red fluorescent protein. 4xLexA is 4 repeats of LexAoperator sequence, LexA is LexA expression activator protein. z-crest3 is islet-1 gene enhancer, making expression of LexA tissue specific. (**b**) $TRPA1_{6His}$ design and ICH staining scheme. HisTag epitope was inserted within the first extracellular loop between S1 and S2 transmembrane helixes of TRPA1. Inderect immunofluorescent staining of cultured cells was performed with monoclonal anti-HisTag antibodies (clone HIS -1, Sigma, H1029) and anti-mouse secondary antibodies Alexa Fluor 488 (Invitrogen, A-21121).

Supplementary figure 2

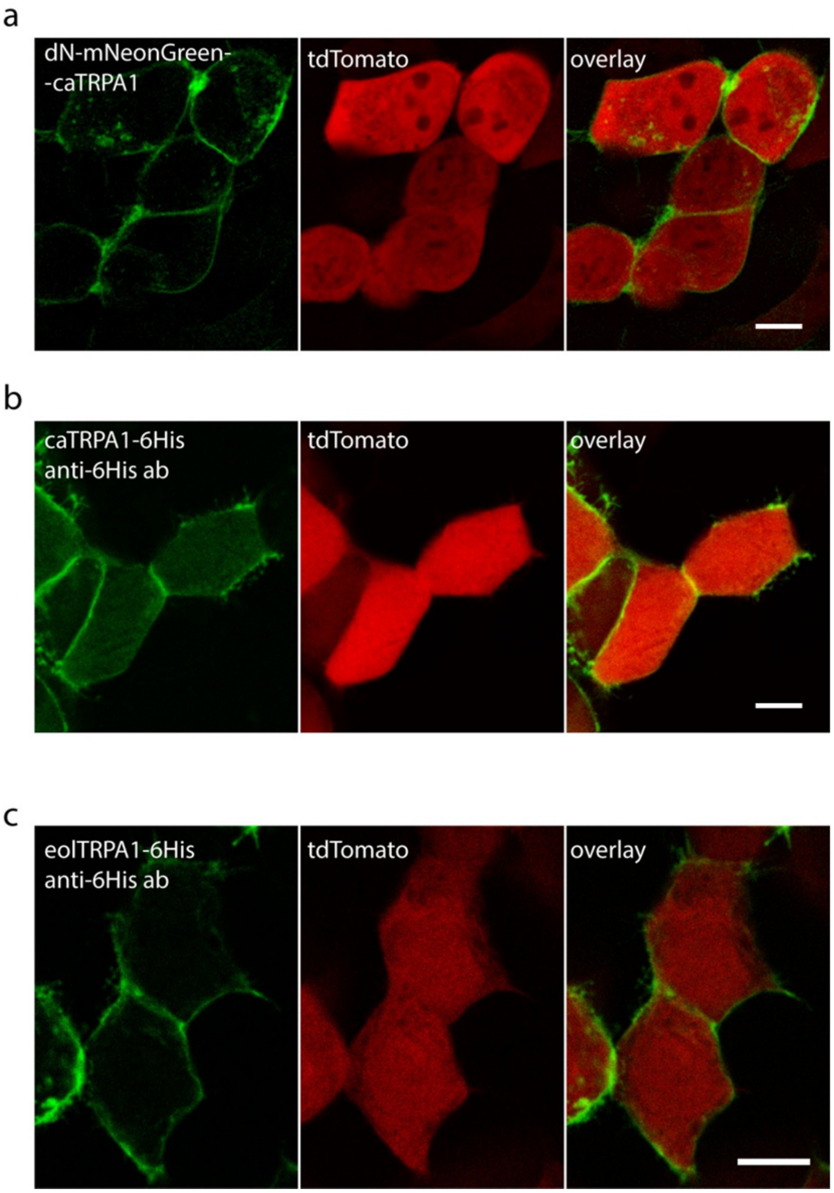

Supplementary Figure 2. Localization of snake TRPA1 channels in HEK293 cells. (**a**) Localization of dN-mNeonGreen-caTRPA1 co-expressed with tdTomato. (**b**) Localization of non-FP labelled caTRPA1 with 6His tag inserted into the peripheral loop (caTRPA1$_{6His}$) visualized using anti-6His antibodies. The channel was co-expressed with tdTomato. (**c**) Localization of non-FP labelled eolTRPA1 with 6His tag inserted into the peripheral loop (eolTRPA1$_{6His}$) visualized using anti-6His antibodies. The channel was co-expressed with tdTomato. All scale bars are 10 µm.

Supplementary figure 3

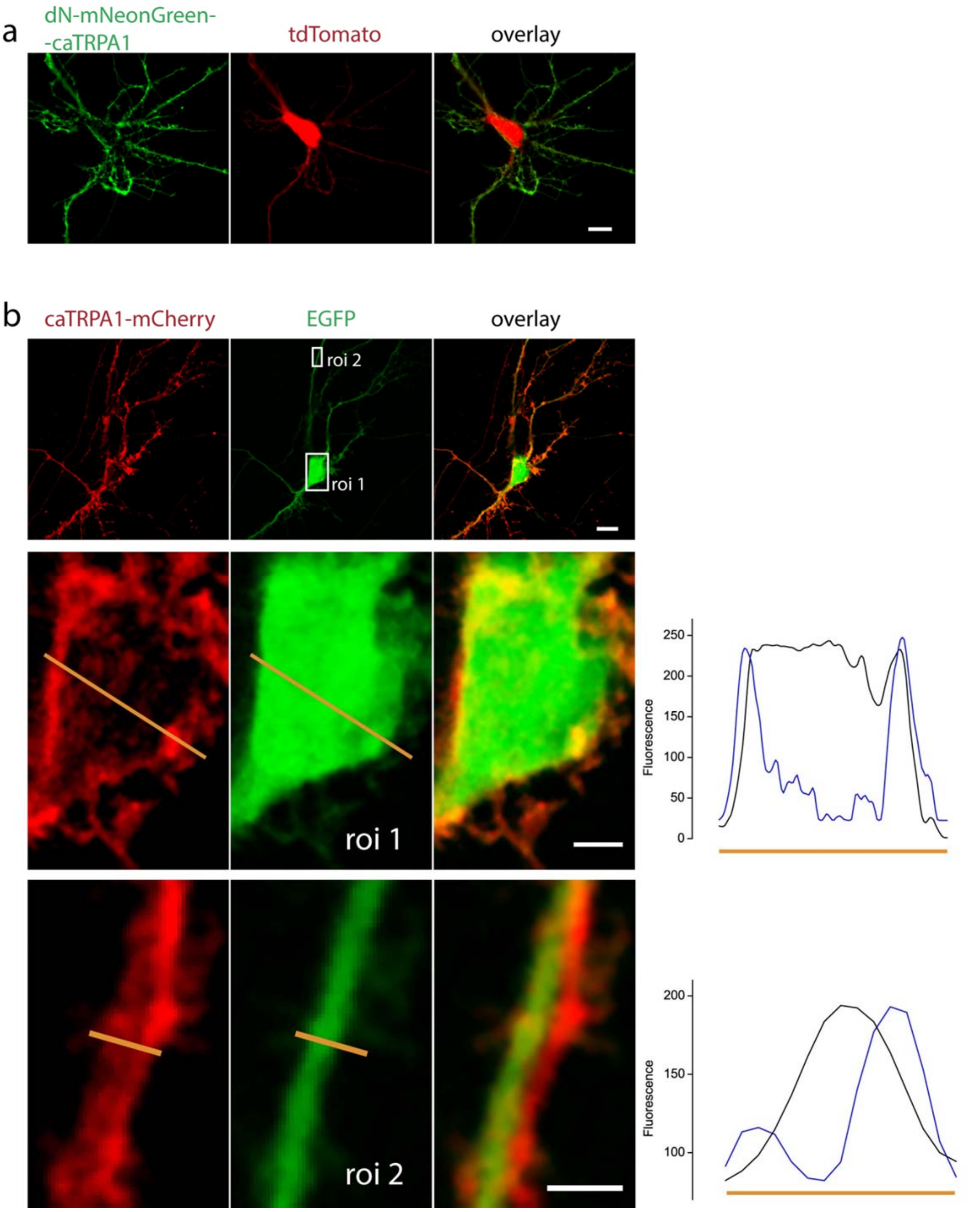

Supplementary Figure 3. Localization of caTRPA1 channels in neurons. (**a**) Localization of dN-mNeonGreen-caTRPA1 co-expressed with tdTomato. Scale bar is 20 µm. (**b**) Localization of caTRPA-mCherry co-expressed with EGFP. Two lower rows show enlarged roi1 and roi2. Line profiles of caTRPA1-mCherry (blue) and EGFP (black) fluorescence along linear roi highlighted by the orange line. Scale bars are 20 µm (upper row) and 5 µm (middle and lower rows).

Supplementary Fig 4

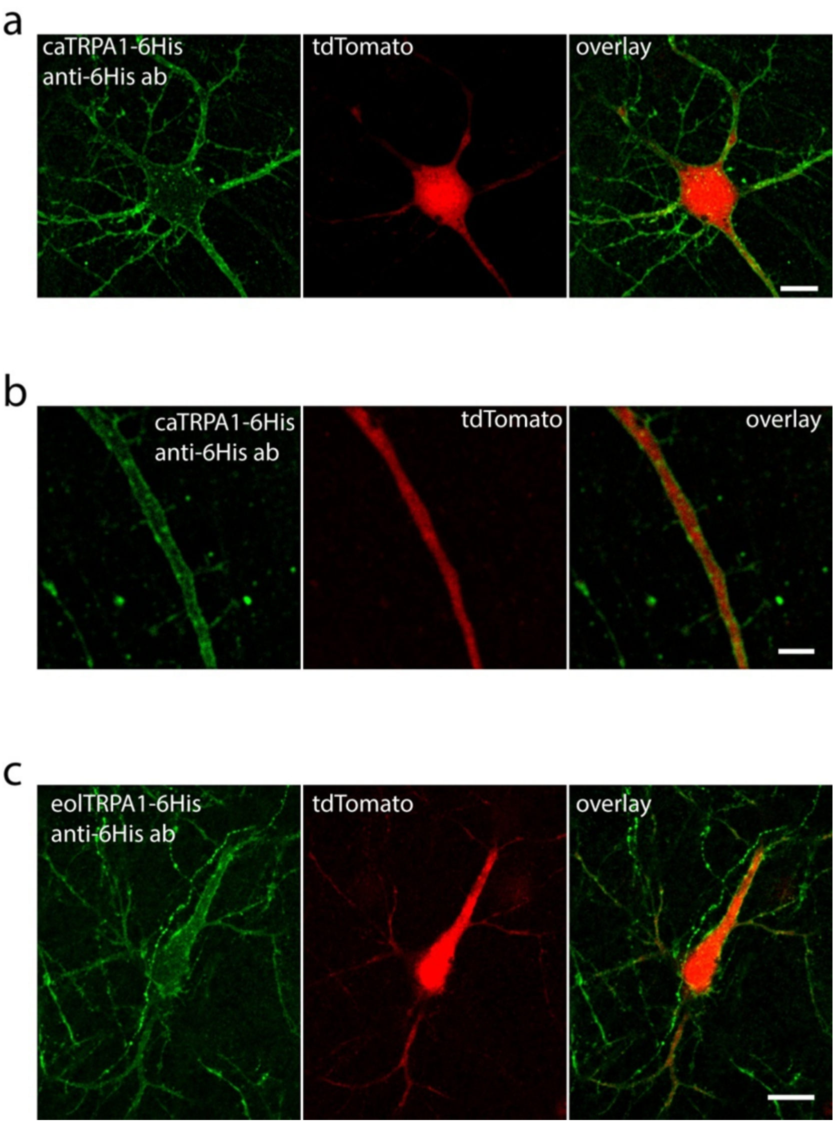

Supplementary Figure 4. Localization of snake TRPA1 channels in neurons (continued). (**a**), (**b**) Localization of caTRPA1$_{6His}$ in cell body (**a**) and process (**b**) of the neuron visualized using anti-6His antibodies. The channel was co-expressed with tdTomato. (**c**) Localization of eolTRPA1$_{6His}$ visualized using anti-6His antibodies. The channel was co-expressed with tdTomato. Scale bars are 10 µm (**a**), 5 µm (**b**) and 20 µm (**c**).

Supplementary figure 5

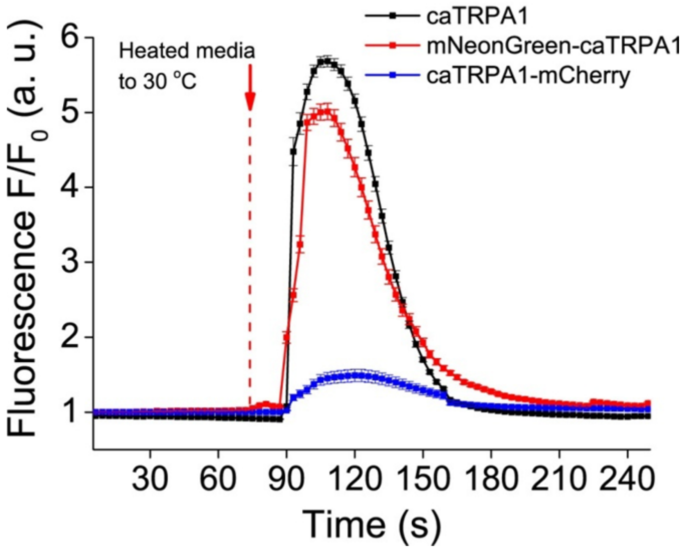

Supplementary Figure 5 caTRPA1 fusions with fluorescent proteins. Comparison of calcium responses of wild type caTRPA1, mNeon-Green-caTRPA1, caTRPA1-mCherry to the addition of media at 30 °C. Temperature control medium temperature was monitored using an electrode thermometer (error ±1 °C).

Supplementary figure 6

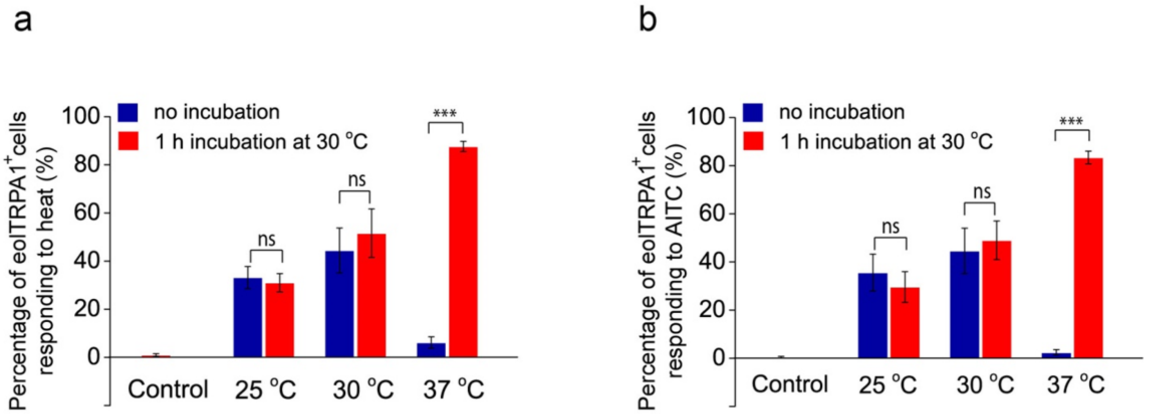

Supplementary Figure 6 Response of HEK293 cells expressing eolTRPA1-IRES2-EGFP and R-GECO1 grown at different temperatures to a rapid elevation of the medium temperature or agonist AITC. (**a**) eolTRPA1 expressing HEK 293 cells were grown at different temperatures and then subjected to heating from 25 °C 38± 1°C with or without pre-incubation at a subthreshold temperature of 30 °C . (**b**) Response of the cells to 200 μM AITC. For each experimental condition 50 or more cells were analyzed from 6 experiments. . ns – not significant. ***p<0.001, paired t-test (two-tailed) for two-group comparisons. The error bars represent the standard errors of mean (SEM).

Supplementary figure 7

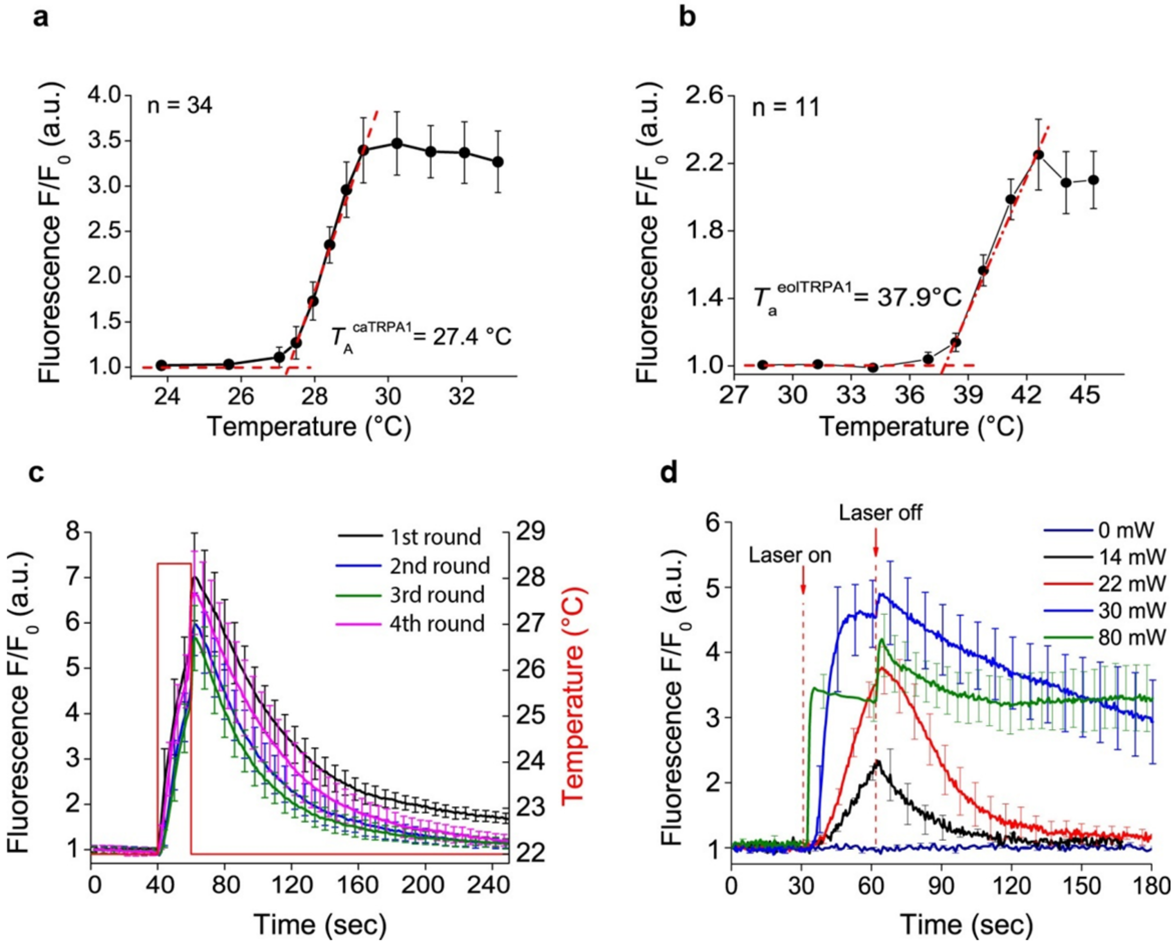

Supplementary Figure 7 Ca^{2+} dynamics in TRPA1-expressing HEK293 cells. (**a**), (**b**) Determination of TRPA1 activation temperature threshold by measuring Ca2+ sensor signal as a function of temperature in the caTRPA1 (*n* = 34) (**a**) and eolTRPA1 (*n* = 11) (**b**) expressing cells. Heating at each point was achieved by 20 sec irradiation of 1440 nm laser, each time starting from the basal temperature 21 °C. (**c**) Four sequential rounds of thermogenic activation of HEK293 cells expressing caTRPA1-IRES-EGFP. Activation events were overlayed and synchronized by the moments of thurning the laser ON. (**d**) Ca^{2+} dynamics in caTRPA1-expressing HEK293 cells depends for different powers of heating quasi-cw laser radiation. Before laser irradiation, the sample was kept at 24 ^{o}C. Each curve is an average over 5 to 11 cells. The error bars represent the standard errors of mean (SEM).

Supplementary figure 8

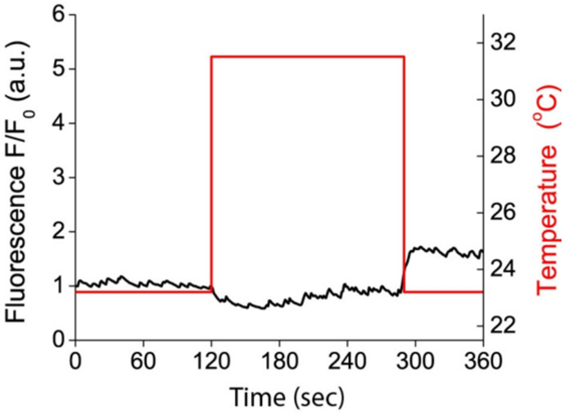

Supplementary figure 8 Stimulation of neurons without TRPA1 does not induce Ca^{2+} transients in the cytoplasm. Neurons were stimulated by the 90-mW, 1440-nm quasi-cw output of the femtosecond OPO for 3 min. Laser irradiation is seen to lead to a slight increase in the basal level of Ca^{2+} right after the turn-off of the laser beam.

Supplementary figure 9

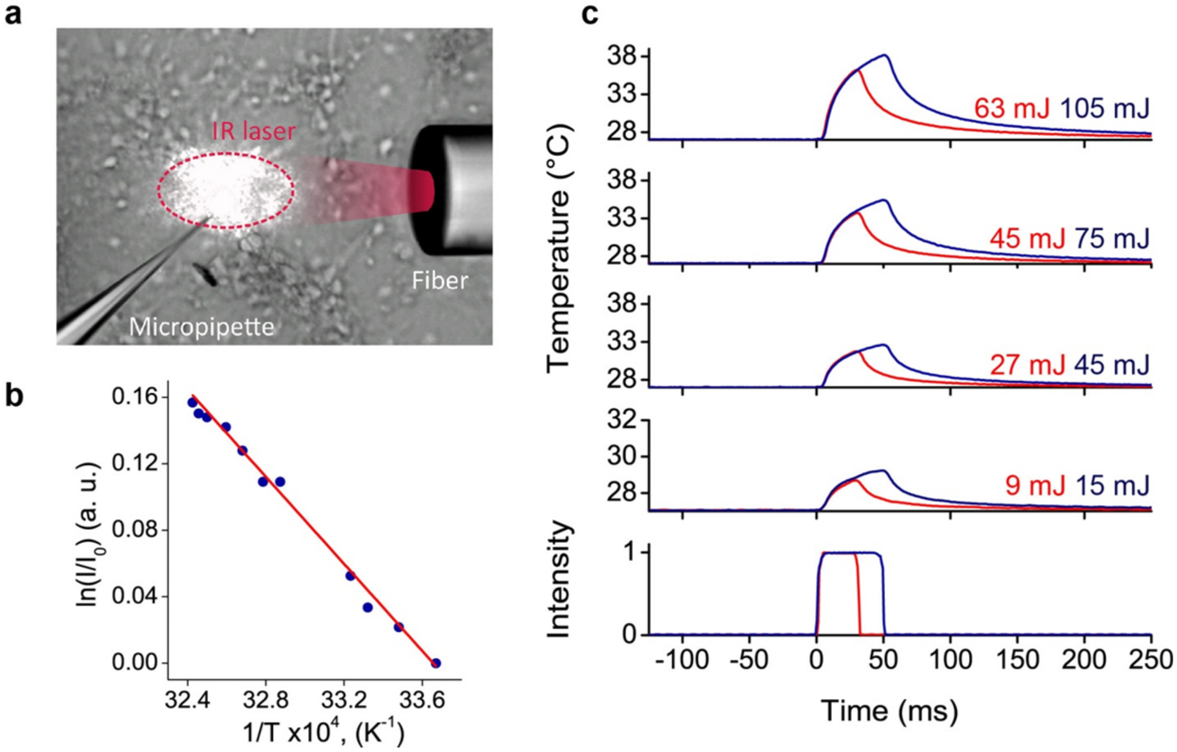

Supplementary figure 9 Local heating and temperature calibration in a culture of neurons. (**a**) Local heating of a neuron in a culture using an optical fiber. The scale bar is 100 µm. The laser spot is visualized by the camera collecting IR light backscattered from the layer of the cells. (**b**) Temperature calibration by temperature-dependent change in the current through a micropipette: (blue circles) experimental data and (red line) the best linear fit. (**c**) Dynamics of local temperature changes induced by 30-ms (red lines) and 50-ms (blue lines) 1050-nm laser pulses of different powers. The laser intensity as a function of time in 30-ms (red lines) and 50-ms (blue lines) pulses of the 1050-nm ytterbium-fiber-laser output is shown in the bottom graph.

Supplementary figure 10

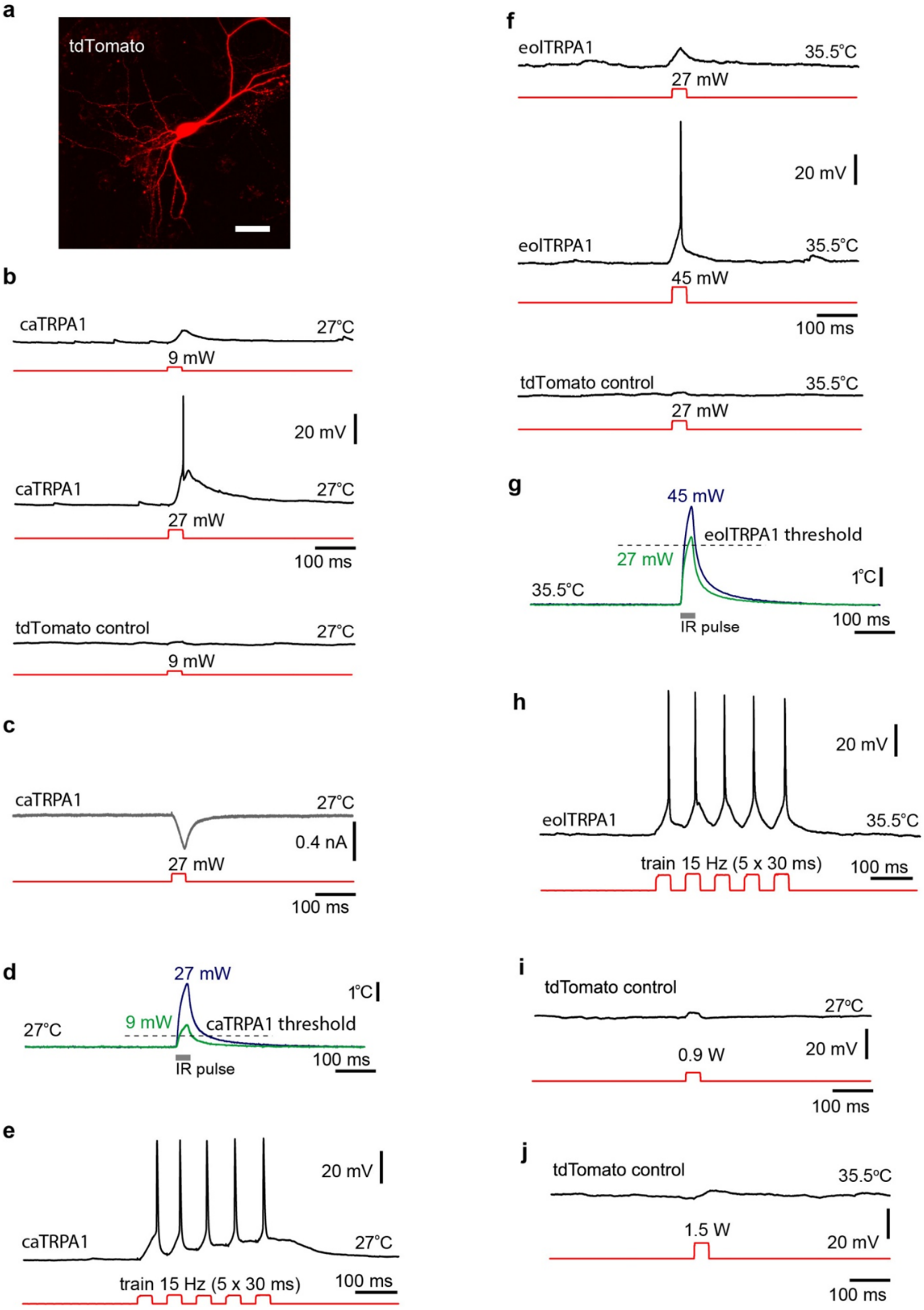

Supplementary figure 10 Electrophysiological study of action potentials and depolarization in neurons expressing caTRPA1 and eolTRPA1 channels stimulated by 30-ms pulses of IR laser radiation. (**a**) A confocal image of a caTRPV1-P2A-tdTomato-expressing neuron. Scale bar is 10 μm. (**b**) Top trace, subthreshold depolarization induced in a caTRPA1+ cultured neuron by a 9-mW IR laser pulse; middle trace, depolarization and action potential in a caTRPA1+ cultured neuron stimulated by a 27-mW IR pulse; bottom trace, control stimulation of a neuron without caTRPA1 channel expression (tdTomato alone) by a 9-mW IR laser pulse. The bath temperature is 27°C. (**c**) The current induced by a 27-mW IR laser pulse in a caTRPA1+ neuron held in voltage-clamp mode at −90 mV. (**d**) Traces of the temperature retrieved, as a part of calibration, from the measurements of the conduction of the patch electrode placed at the center of the area irradiated by 9-mW (green) and 27-mW (blue) laser pulses. The dashed line is the activation threshold of caTRPA1 channels. (**e**) Activity of a caTRPA1+ neuron stimulated with a train of five 30-ms 15-Hz laser pulses (shown in red in the bottom trace). Each laser pulse in the train evokes an action potential. (**f**) Top trace, subthreshold depolarization induced in an eolTRPA1+ cultured neuron by a 27-mW IR laser pulse; middle trace, depolarization and action potential in a eolTRPA1+ cultured neuron stimulated by a 45-mW IR laser pulse; bottom trace, control stimulation of a neuron without eolTRPA1 channel expression (tdTomato alone) by a 27-mW IR laser pulse. The bath temperature is 35.5^{o}C. TRPA1+ and TRPA1- neurons for caTRPA1 and eolTRPA1 were analyzed for IR induced action potential generation with independent t-test, $p < 0.01$. (**g**) Traces of the temperature retrieved, as a part of calibration, from the measurements of the conduction of the patch electrode placed at the center of the area irradiated by 27-mW (green) and 45-mW (blue) laser pulses. The dashed line is the activation threshold of caTRPA1 channels. (**h**) Activity of an eolTRPA1+ neuron stimulated with a train of five 30-ms 15-Hz laser pulses (shown in red in the bottom trace). Each laser pulse in the train evokes an action potential.

Supplementary figure 11

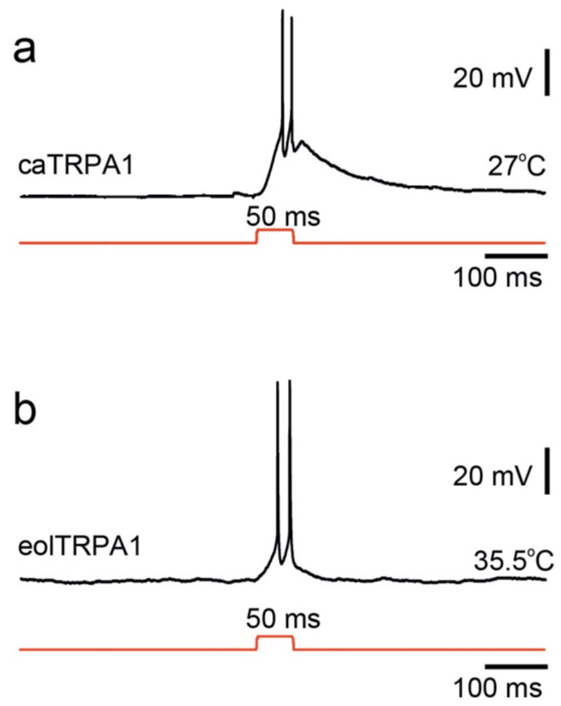

Supplementary figure 11 Electrophysiological study of action potentials and depolarization in neurons expressing caTRPA1 and eolTRPA1 channels stimulated by 50-ms pulses of IR laser radiation. (a) Depolarization and action potential in a caTRPA1+ cultured neuron stimulated by a 27-mW IR pulse. (b) Depolarization and action potential in a eolTRPA1+ cultured neuron stimulated by a 45-mW IR pulse.

Supplementary figure 12

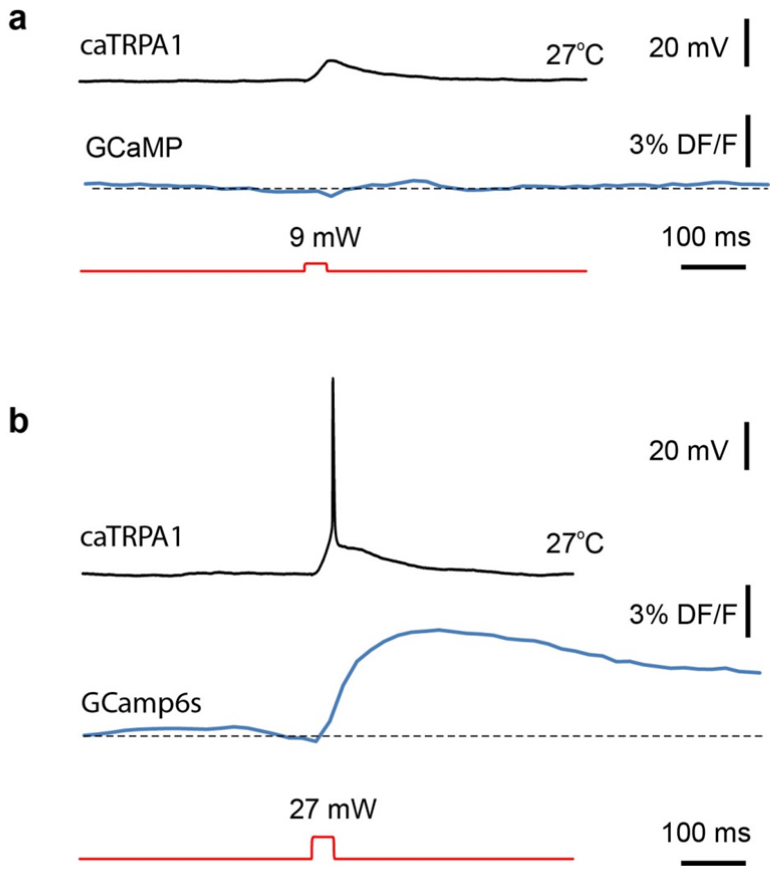

Supplementary figure 12 Electrophysioligical response versus Ca^{2+} dynamics in caTRPA1-P2A-tdTomato-expressing mouse neurons. (**a**) Subthreshold response of a caTRPA1+ cultured neuron stimulated with a 30-ms, 9-mW pulse of 1050-nm laser radiation. The blue trace represents GCaMP6s fluorescence from the somatic region. (**b**) The action potential induced in the same neuron by a 30-ms, 27-mW pulse of 1050-nm laser radiation, leading to Ca^{2+} increase.

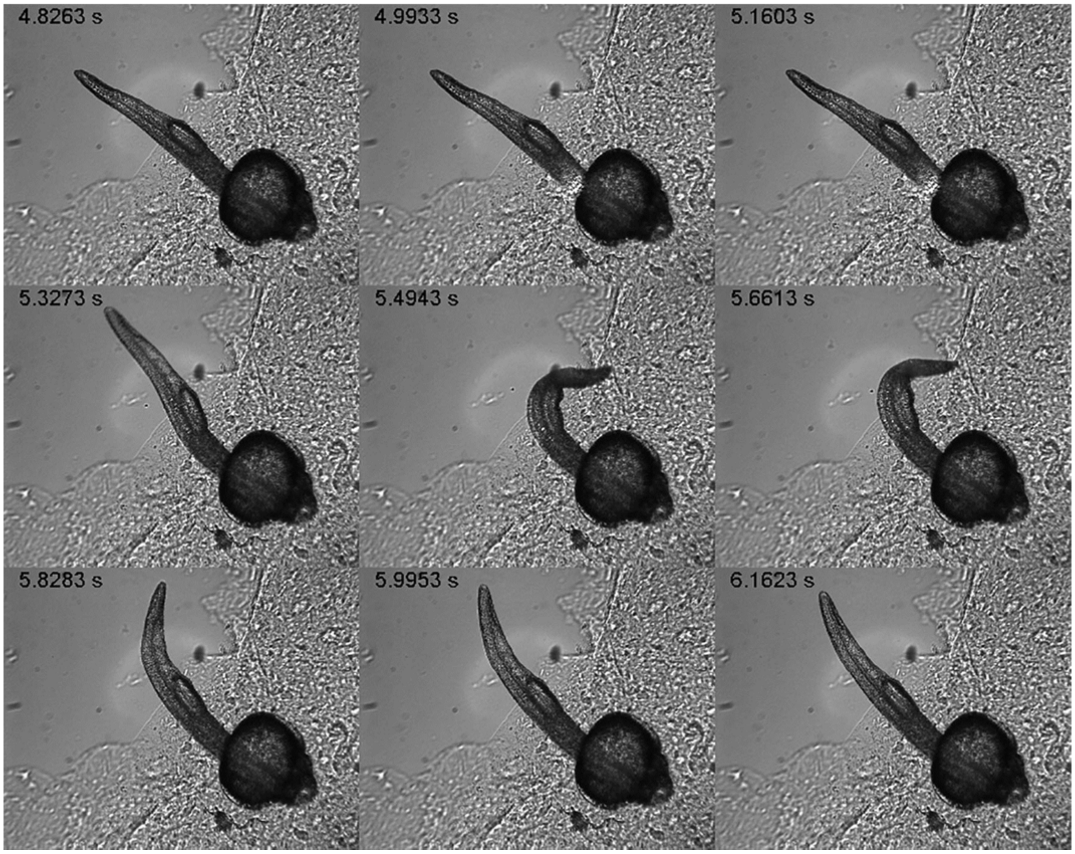

Supplementary Figure 13 Escape behavior induced in 2 dpf zebrafish larvae with 30-mW, 500-ms trains of 100-fs pulses out of the 78-MHz quasi-cw, 1350-nm OPO output.

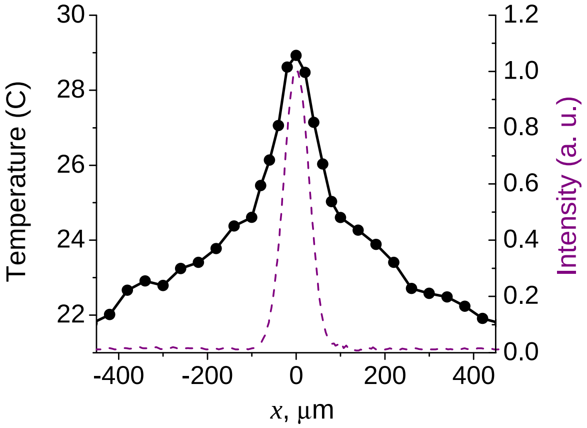

Supplementary figure 14. Spatial profile of temperature distribution (solid line) upon 18 mW 1440 nm laser irradiation measured using 30 μm diamond positioned on the tip of the optical fiber (details in the Methods section). Basal temperature is 19.5°C. Beam width is 60 mm (dashed line).

Supplementary Note 1. Visualization of the channel expression and subcellular targeting

In earlier work[1], YFP fusion was reported for human TRPA1, with N-terminus tagging resulting in functional TRPA1, whereas C-terminal fusions displayed significantly impaired activity. Another study reported that both N- and C- tagged mCherry fusions of *Drosophila melanogaster* dTRPA1 retain both temperature threshold and conductivity[2]. We generated *Crotalus atrox* TRPA1 (caTRPA1) chimeras with fluorescent proteins mCherry[3] and mNeonGreen[4] to visualize Ca^{2+} dynamics in HEK293 cells expressing the chimeric constructs using genetically encoded fluorescent Ca^{2+} probes GCaMP6s and R-GECO 1.1. In the case of C-terminal fusions (caTRPA1-mCherry), rapid heating of cells evoked Ca^{2+} response of dramatically decreased amplitude and speed compared to the wild-type channel (Supplementary Figure 5). Therefore, the C-terminal fusions of caTRPA1 with FPs are not optimal and should be avoided because of the impaired channel activity.

Although caTRPA1-mCherry has somewhat impaired response, it retains native N-terminus which is a main determinant of the channel targeting. Therefore from the localization of the caTRPA1-mCherry we could estimate subcellular distribution pattern of caTRPA1. Figure 1a demonstrates predominantly plasma membrane localization of caTRPA1-mCherry in HEK293 cells indicating proper targeting of the channel. Neuronal expression of caTRPA1-mCherry resulted in mainly plasma membrane targeting of the protein (Supplementary Figure 3b).

Alternatively, we produced an N-terminal fusion of caTRPA1 with mNeonGreen. To ensure proper intracellular trafficking of the construct, we repeated 60 N-terminal amino acids targeting sequence of caTRPA1 on N-terminus of mNeonGreen. The chimeric channel was localized mainly on the plasma membrane of the cells (Supplementary Figure 2a) and demonstrated a stimulation efficiency comparable (80-90%) with that of the wild-type channel (Supplementary Figure 5). Neuronal expression of dN-mNeonGreen-caTRPA1 resulted in predominant PM targeting of the channel (Supplementary Figure 3a). However, the level of mNeonGreen expression in cultured neurons was very low (data not shown). Therefore, we decided against using this method of the TRPA1 channel visualization.

To understand subcellular localization pattern of caTRPA1 we inserted 6-His epitope into a peripheral loop of the channel as it was described earlier for TRPV1[5] and stained HEK293 cells and neurons expressing $caTRPA1_{6His}$-P2A-tdTomato with anti-6His antibodies. The channel localized almost exclusively at the plasma membrane of HEK293 cells (Supplementary Figure 2b) and neurons (Supplementary Figure 4a,b). However, as 6His-tagged version of the sensor demonstrated slower off kinetics (data not shown) in further experiments we used non-tagged version of caTRPA1.

1. Wang, Y.Y., Chang, R.B., Waters, H.N., McKemy, D.D. & Liman, E.R. The nociceptor ion channel TRPA1 is potentiated and inactivated by permeating calcium ions. *J Biol Chem* **283**, 32691-32703 (2008).
2. Vasmer, D., Pooryasin, A., Riemensperger, T. & Fiala, A. Induction of aversive learning through thermogenetic activation of Kenyon cell ensembles in Drosophila. *Front Behav Neurosci* **8**, 174 (2014).

3. Shu, X., Shaner, N.C., Yarbrough, C.A., Tsien, R.Y. & Remington, S.J. Novel chromophores and buried charges control color in mFruits. *Biochemistry* **45**, 9639-9647 (2006).
4. Shaner, N.C. et al. A bright monomeric green fluorescent protein derived from Branchiostoma lanceolatum. *Nature methods* **10**, 407-409 (2013).
5. Stanley, S.A. et al. Radio-wave heating of iron oxide nanoparticles can regulate plasma glucose in mice. *Science* **336**, 604-608 (2012).
